# Reported burden on informal caregivers of ICU survivors: a literature review

**DOI:** 10.1186/s13054-016-1185-9

**Published:** 2016-01-21

**Authors:** Ilse van Beusekom, Ferishta Bakhshi-Raiez, Nicolette F. de Keizer, Dave A. Dongelmans, Marike van der Schaaf

**Affiliations:** 1 Department of Medical Informatics, Academic Medical Center, University of Amsterdam, Amsterdam, The Netherlands; 2 National Intensive Care Evaluation, Amsterdam, The Netherlands; 3 Department of Intensive Care Medicine, Academic Medical Center, University of Amsterdam, Amsterdam, The Netherlands; 4 Department of Rehabilitation, Academic Medical Center, University of Amsterdam, Amsterdam, The Netherlands; 5 Amsterdam School of Health Professions, University of Applied Sciences, Amsterdam, The Netherlands

**Keywords:** Intensive care, Caregivers, Anxiety, Depression, Post-traumatic stress disorder, Follow-up studies

## Abstract

**Background:**

Critical illness and the problems faced after ICU discharge do not only affect the patient, it also negatively impacts patients’ informal caregivers. There is no review which summarizes all types of burden reported in informal caregivers of ICU survivors. It is important that the burdens these informal caregivers suffer are systematically assessed so the caregivers can receive the professional care they need. We aimed to provide a complete overview of the types of burdens reported in informal caregivers of adult ICU survivors, to make recommendations on which burdens should be assessed in this population, and which tools should be used to assess them.

**Method:**

We performed a systematic search in PubMed and CINAHL from database inception until June 2014. All articles reporting on burdens in informal caregivers of adult ICU survivors were included. Two independent reviewers used a standardized form to extract characteristics of informal caregivers, types of burdens and instruments used to assess these burdens. The quality of the included studies was assessed using the Newcastle-Ottawa and the PEDro scales.

**Results:**

The search yielded 2704 articles, of which we included 28 in our review. The most commonly reported outcomes were psychosocial burden. Six months after ICU discharge, the prevalence of anxiety was between 15 % and 24 %, depression between 4.7 % and 36.4 % and post-traumatic stress disorder (PTSD) between 35 % and 57.1 %. Loss of employment, financial burden, lifestyle interference and low health-related quality of life (HRQoL) were also frequently reported. The most commonly used tools were the Hospital Anxiety and Depression Scale (HADS), Centre for Epidemiological Studies-Depression questionnaire, and Impact of Event Scale (IES). The quality of observational studies was low and of randomized studies moderate to fair.

**Conclusions:**

Informal caregivers of ICU survivors suffer a substantial variety of burdens. Although the quality of the included studies was poor, there is evidence that burden in the psychosocial field is most prevalent. We suggest screening informal caregivers of ICU survivors for anxiety, depression, PTSD, and HRQoL using respectively the HADS, IES and Short Form 36 and recommend a follow-up period of at least 6 months.

**Electronic supplementary material:**

The online version of this article (doi:10.1186/s13054-016-1185-9) contains supplementary material, which is available to authorized users.

## Background

Since 1992, the in-hospital mortality of intensive care unit (ICU) patients declined from 32 % [1, 2] to 15–20 % [3, 4]. ICU survivors frequently suffer from psychological distress, reduced social well-being and long-term physical limitations which may result in a reduced quality of life [5]. This combination of complaints has been defined as post-intensive care syndrome (PICS).

PICS and other problems faced after ICU discharge do not only affect the patient, but also reduce the physical, mental, social, and financial position of patients’ informal caregivers, often family members. The combination of psychological problems affecting informal caregivers is known as PICS-family (PICS-F) [6, 7], though there is disagreement on what the term ‘caregiver burden’ entails and how it should be utilized [8].

Systematic reviews have been published on the burden on informal caregivers of ICU patients, but all have different definitions of caregiver burden. Some reviews only include quantitative literature [9], some only focus on the needs and satisfaction of informal caregivers [10, 11] and others focus on specific burdens, such as PICS-F [7], post-traumatic stress disorder (PTSD) [12] or psychosocial burdens [13, 14]. There is no review which summarizes all reported burdens informal caregivers of ICU survivors can suffer after discharge, and no clear overview of tools to asses these burdens. It is important that the burdens on these caregivers, in addition to PICS-F symptoms, are systematically assessed so the informal caregivers can receive professional care if necessary.

We performed a literature review to: (1) assess which burdens on informal caregivers of adult ICU survivors have been documented; (2) assess which assessment tools are used; and (3) make recommendations on which burden should be assessed and which tools could be used.

## Materials and methods

We searched for articles describing burden on informal caregivers of adult ICU survivors, using PubMed and CINAHL from database inception to June 2014. The search strategy is presented in Table 1. Only English and Dutch articles were included.

**Table 1:** Search strategy

Database		Search terms	
PubMed	Participant	Mesh	Caregivers; family; spouses; family health; proxy
	ICU	Mesh	Critical care; critical illness; intensive care units; intensive care
	Exclusion	Mesh	Intensive care, neonatal; intensive care units, pediatric; intensive care units, neonatal; child; infant; infant, newborn; child, preschool
CINAHL	Participant	Mesh	Family; caregiver burden; caregivers; spouses; family health
	ICU	Mesh	Critical care; critical illness; intensive care units
	Exclusion	Mesh	Intensive care, neonatal; intensive care units, pediatric; intensive care units, neonatal; neonatal intensive care nursing; pediatric critical care nursing; child; infant; infant, newborn; child, preschool

Two authors (IvB and FBR) independently assessed the titles and abstracts of 50 randomly selected articles to ensure that the inclusion criteria were not ambiguous. For 47 (94 %) of these articles the inclusion criteria were applied identically. After discussing the differences, consensus was reached. We considered the consistency between the two authors sufficient and made no alterations to the inclusion criteria. We included original studies if: the subject of the study was an informal caregiver of an adult ICU patient; the ICU patient was discharged from hospital alive; at least one of the measurements of the burden took place after hospital discharge; and the burden on the informal caregiver was a main outcome of the study. We excluded studies on deceased ICU patients, studies on the needs or satisfaction of the informal caregiver, presence during cardiopulmonary resuscitation, and involvement in end-of-life decisions, because we hypothesized that informal caregivers of these groups would suffer different burdens.

One author (IvB) evaluated the titles and abstracts of all articles. The abstracts were either included, excluded or marked as doubtful. Another author (FBR) read the title and abstract of articles marked as doubtful and both authors discussed these articles to reach consensus on inclusion. We supplemented our searches by scanning the reference lists of previously included articles. The full text of all eligible articles was read by two authors (IvB and one of FBR, NdK, MvdS, or DAD). Both authors extracted data on the study type, characteristics of the informal caregivers, hospital and setting, type of burden and instruments used to assess the burden. If information could not be extracted from the article or online appendices, we e-mailed the corresponding author for additional information. We assessed the quality of the quantitative articles, using the Newcastle-Ottawa scale (NOS) [15] for observational studies and the PEDro scale [16] for randomized trials.

## Results

**Fig. 1:**
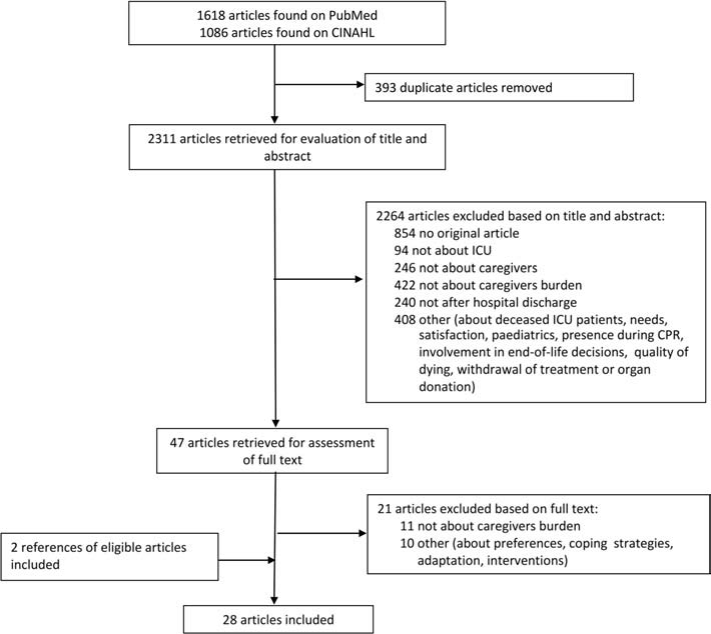
Flow diagram of literature search results, the inclusion process and the reason for exclusion

We retrieved 2704 articles using the search strategy described in Table 1. After removing duplicates, we assessed the title and abstract of 2311 articles and excluded 2264 articles based on title and abstract. Figure 1 summarizes the inclusion process and provides the reasons for exclusion. We assessed the full text of 47 articles and excluded another 21 articles. We hand searched the references of the 26 included articles and included two additional studies. Nine authors were contacted to complete the data for 12 articles and six authors responded.

### Study characteristics and quality

There is a wide variety in study and informal caregiver characteristics (Table S1 in Additional file 1). Fourteen studies were conducted in northern America [17–30], 12 in Europe [31–42], one in Australia [43] and one in Botswana [44]. The follow-up period ranged from 2 weeks after hospital discharge to 4 years after ICU discharge. Most of the informal caregivers were female (47–100 %) and most of them were the partner/spouse of the ICU survivor (24–100 %).

We present the results of the quality assessment of the included articles in Table 2. The NOS scores for the 24 observational studies ranged from two to three on a scale of zero to nine, indicating low quality. The PEDro scale score for the three randomized controlled trials ranged from four to seven on a scale of zero to ten, indicating moderate to fair quality.

**Table 2:** Quality of included studies

Non-randomized studies assessed with the Newcastle-Ottawa Scale				
Author, year	Selection	Comparability	Outcome	Total score
Ågård, 2014 [31]	1	0	2	3
Anderson, 2008 [17]	1	0	2	3
Azoulay, 2005 [32]	1	0	2	3
Bayen, 2013 [33]	1	0	2	3
Cameron, 2006 [18]	1	0	2	3
Choi, 2011 [19]	1	0	1	2
Choi, 2012 [20]	1	0	2	3
Dithole, 2013 [44]	1	0	1	2
Douglas, 2003 [22]	1	0	2	3
Douglas, 2010 [24]	1	0	2	3
Foster, 2003 [43]	1	0	2	3
Garrouste-Orgeas, 2012 [34]	1	0	2	3
Im, 2004 [25]	1	0	2	3
Lemiale, 2010 [37]	1	0	1	2
De Miranda, 2011 [38]	1	0	2	3
McAdam, 2012 [26]	1	0	2	3
Myhren, 2004 [39]	1	0	1	2
Van Pelt, 2007 [27]	1	0	2	3
Van Pelt, 2010 [28]	1	0	1	2
Rodríguez, 2005 [41]	1	0	1	2
Rodríguez, 2005 [40]	1	0	1	2
Swoboda, 2002 [29]	1	0	2	3
Wartella, 2009 [30]	1	0	1	2
Young, 2005 [42]	1	0	2	3
Randomized controlled trails assessed with the PEDro Scale				
Author, year		Total score		
Douglas, 2005 [23]		4/10		
Jones, 2004 [36]		7/10		
Jones, 2012 [35]		5/10		

### Burden

We found a large diversity in types of burden reported. Table 3 shows a summary of the main findings. A complete overview of all types of burden is presented in Table S2 in Additional file 2.

**Table 3:** Summary of main findings of the reported burden

Type of burden	Time of measurement	Reported outcomes
Anxiety^a^	During admission	42–80 %
	3 months	24–63 %
	6 months	15–24 %
Depression	During admission	16–90 %
	3 months	12–26 %
	6 months	5–36 %
	12 months	23–44 %
Post-traumatic stress disorder	During admission	57 %
	3 months	30–42 %
	6 months	35–57 %
	12 months	32–80 %
Employment status	Up to 50 % of the informal caregivers reduced their work hours, quit their job or were fired in order to provide informal care	
Health-related quality of life	Major decreases in mental health, limited changes in physical health	
Use of medication	Between 8 % and 32 % of informal caregivers started to use medications after the ICU admittance of their relative	
Lifestyle interference	Up to 12 months after discharge, almost 50 % of informal caregivers had to quit activities in order to take care of the patient	

^a^Anxiety was not assessed at 12 months after discharge

Nineteen studies (68 %) assessed depression (Table S3 in Additional file 3), of these eight used the Hospital Anxiety and Depression Scale (HADS) subscale [17, 26, 32, 34, 36–38, 42], seven the Centre for Epidemiological Studies-Depression (CES-D) questionnaire [18, 22–25, 27, 28, 31], one the short version of the CES-D [20], one the Zarit Burden Inventory [33], one the Brief Symptom Inventory (BSI) [30] and one a self-developed questionnaire [39]. The prevalence was between 16 % and 90 % during ICU or hospital stay and between 12.2 % and 26.2 % 3 months, 4.7 % and 36.4 % 6 months, and 22.8 % and 44 % 12 months after ICU discharge. The cross-sectional study reported a prevalence of 31.9 %.

Ten studies assessed anxiety (36 %) (Table S4 in Additional file 4), of these eight used the HADS [17, 26, 32, 34, 36–38, 42], one the BSI [30] and one a self-developed questionnaire [39]. The prevalence was between 42 % and 79.7 % during ICU or hospital stay and between 24.4 % and 62.5 % 3 months and 15 % and 24 % 6 months after ICU discharge.

Post-traumatic stress was assessed in eight studies (29 %) (Table S5 in Additional file 5), of these three used the Impact of Event Scale (IES) [17, 32, 36], three the IES-Revised (IES-R) [26, 34, 38], one the PTSD Checklist-specific scale [44] and one the Post-Traumatic Stress Syndrome-14 screening tool [35]. The prevalence was 56.8 % during ICU stay and between 29.8 % and 42 % 3 months, 35 % and 57.1 % 6 months and 31.7 % to 80 % 12 months after ICU discharge.

Thirteen studies described informal caregivers’ employment status [18–20, 22–25, 27, 29, 31, 32, 43, 44] and at study enrolment between 25.4 % and 72.3 % were in paid employment. Four studies reported a reduction in employment around 2 months after enrolment [24, 25, 27, 29] and two reported that almost 50 % of caregivers, who had been employed at enrolment, reduced their work hours, quit their job or were fired in order to provide informal care [24, 29].

Of the informal caregivers, who were employed prior to the ICU admission, 84.6 % had returned to their previous work 12 months after enrolment [31]. Their mean sick leave was 11 days (range 4–42) for full-time employees and 9 days (range 0–44) for part-time employees during the patient’s ICU stay and 17 days (range 0–124) for full-time employees and 21 days (range 0–106) for part-time employees during the 12 months after ICU discharge [31]. Thirty-eight percent of the informal caregivers reported that it was somewhat difficult to pay for basic needs such as food, housing, medical care and heating. Some of them even moved to a less expensive home, delayed educational plans or medical care for themselves or another family member, or filed for bankruptcy due to the financial burdens [29].

Of the seven studies which described health-related quality of life (HRQoL), four used the Short Form 36 (SF-36) [18, 32, 33, 37], one used the Short Form 8 (SF-8) [23] and two used a single-measure item [22, 24] (Table S6 in Additional file 6). Two found no change in physical health scores [33, 37], one reported that 36 % of informal caregivers experienced negative changes in their physical health [22], and one reported no statistically significant differences in changes in physical health between informal caregivers and controls over time [23]. Three studies reported major decreases in the mental health of informal caregivers [32, 33, 37], one reported that informal caregivers scored lower on all domains of the SF-36 than an age- and gender-matched population [18], and one reported a slight decrease in general health [32].

Six studies reported on informal caregivers’ use of antidepressant, anxiolytic, hypnotic and psychotropic medication [24, 32, 37–39, 44]. Between 8.4 % and 32 % of informal caregivers started to use these medications after ICU admittance [32, 37, 38] and 14 % used more hypnotics and 4 % more anxiolytics after the ICU stay than before [39]. Between 8.4 % [32] and 17 % [38] of informal caregivers received psychiatric or psychological support after their relative’s ICU admission, 40 % saw a healthcare professional for emotional problems [20]. Six months after ICU admission, 21.1 % had delayed obtaining care for themselves because of the patient’s illness [29].

Eight studies assessed the lifestyle interference of informal caregivers (Table S7 in Additional file 7). Two used the Activity Restriction Scale [27, 28], two the Changes in Role Function scale [19, 25], one the Caregiving Impact Scale [18], one the “objective indicator” portion of the “objective and subjective burden” tool [22], one the Family Impact Survey [29] and one qualitative methods [42]. Lifestyle interference was high [19, 27, 28], the percentage of informal caregivers who had quit other activities in order to care for the ICU survivor was 84.5 % 1 month and 45.8 % 12 months after ICU admission [29]. One month after ICU discharge, 75 % had moderate or great restrictions in visiting friends and 48 % in practicing hobbies and recreation [19]. They provided about 5 hours of care a day [22, 25, 27, 43] between hospital discharge [22] and 12 months after initiation of mechanical ventilation [27].

### Qualitative research

Five studies had qualitative elements. One relied entirely on semi-structured interviews [21] and four had some qualitative components [31, 38, 42, 44]. They mainly reported psychosocial burdens, such as sleep disorders, nightmares, sadness, distress, anxiety, exhaustion, crying for no apparent reason and keeping a distance from family and friends. Parents described it as ‘emotionally draining’ to explain the situation to the children [21] or were scared of leaving children alone with the ICU survivor at home. Children’s involvement made it more complicated to balance the logistics of home life and work [21, 31]. An ICU admission can also impact the relationship between the ICU survivor and the informal caregiver. Informal caregivers and ICU survivors can feel more irritated with each other, experience less freedom than before [42], experience a sense of increased distance in their relationship [21] or even attribute the end of their relationship to the ICU admission [31]. However, one couple stated that they showed each other more tenderness and respect and another reported that their life was more positive following the ICU admission [42].

## Discussion

We performed a literature review to assess the burdens experienced by informal caregivers of adult ICU survivors have been documented, how they are assessed and to make recommendations on which burdens should be assessed. We have shown that informal caregivers of ICU survivors have extensive burdens following the patient’s ICU admission. This is reflected in psychosocial status, quality of life, lifestyle, employment and financial status. The most frequently used assessment tools were the HADS, the CES-D, the IES and the SF-36.

Psychosocial burdens are most commonly reported and, in this review, we described these in depth. Generally, the prevalence was highest during and shortly after the ICU admission, decreased over time, but remained higher compared to control groups. In contrast, the prevalence of PTSD increased over time. Although different measurement tools were used, the prevalence of depression among informal caregivers of ICU survivors was higher than among informal caregivers of patients with colorectal cancer [45] and following coronary bypass surgery [46], stroke, hip fracture, congestive heart failure and myocardial infarction [46]. We found that, 3 months after ICU discharge, between a quarter and two-thirds of informal caregivers reported anxiety. This is similar to the prevalence reported in a systematic review on anxiety in informal caregivers of people with dementia [47]. Burdens such as insomnia, concentration problems, fear of death and spiritual problems were only described by few authors in low-quality, observational studies. However, these burdens can influence informal caregivers substantially. Further research on the scope of these problems and the appropriated assessment tools is necessary.

A range of assessment tools can be used to quantify the burdens on informal caregivers. However, these tools use different cut-off points to quantify the burden. For example, the HADS uses two different cut-off points. Scores of eight to ten on the anxiety or depression subscale potentially indicate pathology and scores of 11 or more are considered more definite [48]. However, these tools can be used as screening instruments, but are not valid methods for obtaining a clinical diagnosis and cannot predict which informal caregivers will need professional treatment to recover.

Correct use of the questionnaires is crucial, but not always found. For example in the article by McAdam [26] the IER-R is used for informal caregivers during ICU admission of their relative and refers to the outcome as PTSD. However, according to the definition of PTSD, PTSD cannot be evaluated during the event. Symptoms have to be present for at least 1 month after the event of interest in order to be diagnosed as PTSD [49].

Possible benefits of post-ICU clinics for ICU survivors are mentioned before [50]. However, we did not find any recommendations on screening informal caregivers in post-ICU care, though there are recommendations on inviting the informal caregiver to the patient’s post-ICU care [51]. Considering the high prevalence of a wide range of burdens in informal caregivers, we highly recommend assessing the informal caregiver as part of the post-ICU care so they can be referred to the appropriate healthcare provider(s) if necessary.

There is a large resemblance between a recently published systematic review about the psychosocial outcomes informal caregivers of ICU patients can suffer [13] and our study, as 11 articles were included in both studies. However, a strength of our study is that we did not restrict our literature search to psychosocial outcomes and could include 17 additional articles [17, 26, 29–34, 36–44]. Consequently, we also report on other burdens such as anxiety, loss of employment, financial problems and healthcare consumption. Recognition of these additional types of burden is important for referral to the appropriated healthcare provider.

Another strength of our review is that we included both quantitative and qualitative studies describing burdens informal caregivers can suffer. This means that we could identify additional burdens such as sleeping disorders and negative impacts on social life and relationships [21, 31, 38, 42, 44].

Our study also has some limitations. Two pairs of articles describe the same data from samples of 57 [40, 41] and 284 [32, 37] informal caregivers. Both pairs of articles report on results obtained using the same instruments at the same time points. Since we did not perform a meta-analysis, we believe that the influence of these duplicate data is limited. In addition, the methodological quality of the 24 observational studies was low and the three randomized studies moderate to fair. Although all of the studies report similar results, more high-quality studies are needed to obtain accurate assessments of the prevalence and severity of burdens informal caregivers suffer.

## Conclusions

Our findings suggest that critical illness and problems faced after ICU discharge have long-term effects on informal caregivers of ICU survivors. Psychosocial symptoms of PICS-F, such as depression, anxiety and post-traumatic stress symptoms, and decreased health-related quality of life are the most commonly reported burden.

We recommend screening for these burdens and recommend a follow-up period of at least 6 months. Screening could be done by the ICU department or rehabilitation department of the hospital where the patient was admitted. Screening on symptoms of PICS-F could be integrated in the post-ICU care, if offered, for ICU patients. The screening could be performed with a telephone consultation, or as part of a visit to a post-ICU clinic by the ICU survivor [51]; thus combining aftercare for patients and their informal caregivers. In the absence of an ICU aftercare programme, it is important that the family physician should be aware of the risk for PICS-F symptoms in informal caregivers of former ICU patients. Informal caregivers can be screened using validated tools such as the HADS, IES, CES-D and SF-36.

## Additional files

Additional file 1: Table S1. Informal caregiver characteristics. (DOC 206 kb)

Additional file 2: Table S2. Overview of all types of burden reported in the included articles. (DOC 528 kb)

Additional file 3: Table S3. Depression: assessment tools, time points and outcomes measures for caregivers for quantitative studies. (DOC 179 kb)

Additional file 4: Table S4. Anxiety: assessment tools, time points and outcomes measures for caregivers for quantitative studies. (DOC 93 kb)

Additional file 5: Table S5. Post-traumatic stress: assessment tools, time points and outcomes measures for caregivers for quantitative studies. (DOC 77 kb)

Additional file 6: Table S6. Health-related quality of life: assessment tools, time points and outcomes measures for caregivers for quantitative studies. (DOC 80 kb)

Additional file 7: Table S7. Lifestyle interference: assessment tools, time points and outcomes measures for caregivers for quantitative studies. (DOC 65 kb)

## Abbreviations

BSI: Brief Symptom Inventory
CES-D: Centre for Epidemiological Studies-Depression questionnaire
HADS: Hospital Anxiety and Depression Scale
HRQoL: Health-related quality of life
ICU: Intensive care unit
IES: Impact of Event Scale
NOS: Newcastle-Ottawa Scale
PICS: Post-intensive care syndrome
PICS-F: Post-intensive care syndrome-family
PTSD: Posttraumatic stress disorder
SF-36: Short Form 36

## References

[1] Goldhill DR, Sumner A (1998). Outcome of intensive care patients in a group of British intensive care units. Crit Care Med.

[2] Moreno R, Morais P (1997). Outcome prediction in intensive care: results of a prospective, multicentre. Portuguese study. Intensive Care Med.

[3] Kasza J, Moran JL, Solomon PJ (2013). Outcome AN-ANZICSCf, Resource Evaluation C. Evaluating the performance of Australian and New Zealand intensive care units in 2009 and 2010. Stat Med.

[4] Brinkman S, de Jonge E, Abu-Hanna A, Arbous MS, de Lange DW, de Keizer NF (2013). Mortality after hospital discharge in ICU patients. Crit Care Med.

[5] van der Schaaf M, Beelen A, Dongelmans DA, Vroom MB, Nollet F (2009). Functional status after intensive care: a challenge for rehabilitation professionals to improve outcome. J Rehabil Med.

[6] Needham DM, Davidson J, Cohen H, Hopkins RO, Weinert C, Wunsch H (2012). Improving long-term outcomes after discharge from intensive care unit: report from a stakeholders’ conference. Crit Care Med.

[7] Davidson JE, Jones C, Bienvenu OJ (2012). Family response to critical illness: postintensive care syndrome-family. Crit Care Med.

[8] Bastawrous M (2013). Caregiver burden--a critical discussion. Int J Nurs Stud.

[9] Kentish-Barnes N, Lemiale V, Chaize M, Pochard F, Azoulay E (2009). Assessing burden in families of critical care patients. Crit Care Med.

[10] Paul F, Rattray J (2008). Short- and long-term impact of critical illness on relatives: literature review. J Adv Nurs.

[11] Verhaeghe S, Defloor T, Van Zuuren F, Duijnstee M, Grypdonck M (2005). The needs and experiences of family members of adult patients in an intensive care unit: a review of the literature. J Clin Nurs.

[12] Kross EK, Gries CJ, Curtis JR (2008). Posttraumatic stress disorder following critical illness. Crit Care Clin.

[13] Haines KJ, Denehy L, Skinner EH, Warrillow S, Berney S (2015). Psychosocial outcomes in informal caregivers of the critically ill: a systematic review. Crit Care Med.

[14] McAdam JL, Puntillo K (2009). Symptoms experienced by family members of patients in intensive care units. Am J Crit Care.

[15] Wells G OCD, Peterson J, Welch V, Losos M, Tugwell P. The Newcastle-Ottawa Scale (NOS) for assessing the quality of nonrandomised studies in meta-analysis. http://www.ohri.ca/programs/clinical_epidemiology/oxford.asp. Accessed 22 January 2015.

[16] Maher CG, Sherrington C, Herbert RD, Moseley AM, Elkins M (2003). Reliability of the PEDro scale for rating quality of randomized controlled trials. Phys Ther.

[17] Anderson WG, Arnold RM, Angus DC, Bryce CL (2008). Posttraumatic stress and complicated grief in family members of patients in the intensive care unit. J Gen Intern Med.

[18] Cameron JI, Herridge MS, Tansey CM, McAndrews MP, Cheung AM (2006). Well-being in informal caregivers of survivors of acute respiratory distress syndrome. Crit Care Med.

[19] Choi J, Donahoe MP, Zullo TG, Hoffman LA (2011). Caregivers of the chronically critically ill after discharge from the intensive care unit: six months’ experience. Am J Crit Care.

[20] Choi J, Sherwood PR, Schulz R, Ren D, Donahoe MP, Given B (2012). Patterns of depressive symptoms in caregivers of mechanically ventilated critically ill adults from intensive care unit admission to 2 months postintensive care unit discharge: a pilot study. Crit Care Med.

[21] Cox CE, Docherty SL, Brandon DH, Whaley C, Attix DK, Clay AS (2009). Surviving critical illness: acute respiratory distress syndrome as experienced by patients and their caregivers. Crit Care Med.

[22] Douglas SL, Daly BJ (2003). Caregivers of long-term ventilator patients: physical and psychological outcomes. Chest.

[23] Douglas SL, Daly BJ, Kelley CG, O’Toole E, Montenegro H (2005). Impact of a disease management program upon caregivers of chronically critically ill patients. Chest.

[24] Douglas SL, Daly BJ, O’Toole E, Hickman RL (2010). Depression among white and nonwhite caregivers of the chronically critically ill. J Crit Care.

[25] Im K, Belle SH, Schulz R, Mendelsohn AB, Chelluri L, Investigators Q-M (2004). Prevalence and outcomes of caregiving after prolonged (> or =48 hours) mechanical ventilation in the ICU. Chest.

[26] McAdam JL, Fontaine DK, White DB, Dracup KA, Puntillo KA (2012). Psychological symptoms of family members of high-risk intensive care unit patients. Am J Crit Care.

[27] Van Pelt DC, Milbrandt EB, Qin L, Weissfeld LA, Rotondi AJ, Schulz R (2007). Informal caregiver burden among survivors of prolonged mechanical ventilation. Am J Respir Crit Care Med.

[28] Van Pelt DC, Schulz R, Chelluri L, Pinsky MR (2010). Patient-specific, time-varying predictors of post-ICU informal caregiver burden: the caregiver outcomes after ICU discharge project. Chest.

[29] Swoboda SM, Lipsett PA (2002). Impact of a prolonged surgical critical illness on patients’ families. Am J Crit Care.

[30] Wartella JE, Auerbach SM, Ward KR (2009). Emotional distress, coping and adjustment in family members of neuroscience intensive care unit patients. J Psychosom Res.

[31] Agard AS, Lomborg K, Tonnesen E, Egerod I (2014). Rehabilitation activities, out-patient visits and employment in patients and partners the first year after ICU: a descriptive study. Intensive Crit Care Nurs.

[32] Azoulay E, Pochard F, Kentish-Barnes N, Chevret S, Aboab J, Adrie C (2005). Risk of post-traumatic stress symptoms in family members of intensive care unit patients. Am J Respir Crit Care Med.

[33] Bayen E, Pradat-Diehl P, Jourdan C, Ghout I, Bosserelle V, Azerad S (2013). Predictors of informal care burden 1 year after a severe traumatic brain injury: results from the PariS-TBI study. J Head Trauma Rehabil.

[34] Garrouste-Orgeas M, Coquet I, Perier A, Timsit JF, Pochard F, Lancrin F (2012). Impact of an intensive care unit diary on psychological distress in patients and relatives*. Crit Care Med.

[35] Jones C, Backman C, Griffiths RD (2012). Intensive care diaries and relatives’ symptoms of posttraumatic stress disorder after critical illness: a pilot study. Am J Crit Care.

[36] Jones C, Skirrow P, Griffiths RD, Humphris G, Ingleby S, Eddleston J (2004). Post-traumatic stress disorder-related symptoms in relatives of patients following intensive care. Intensive Care Med.

[37] Lemiale V, Kentish-Barnes N, Chaize M, Aboab J, Adrie C, Annane D (2010). Health-related quality of life in family members of intensive care unit patients. J Palliat Med.

[38] de Miranda S, Pochard F, Chaize M, Megarbane B, Cuvelier A, Bele N (2011). Postintensive care unit psychological burden in patients with chronic obstructive pulmonary disease and informal caregivers: a multicenter study. Crit Care Med.

[39] Myhren H, Ekeberg O, Langen I, Stokland O (2004). Emotional strain, communication, and satisfaction of family members in the intensive care unit compared with expectations of the medical staff: experiences from a Norwegian University Hospital. Intensive Care Med.

[40] Rodríguez AM (2005). Psychosocial adaptation in relatives of critically injured patients admitted to an intensive care unit. Span J Psychol.

[41] Rodríguez AM, Gregorio MA, Rodriguez AG (2005). Psychological repercussions in family members of hospitalised critical condition patients. J Psychosom Res.

[42] Young E, Eddleston J, Ingleby S, Streets J, McJanet L, Wang M (2005). Returning home after intensive care: a comparison of symptoms of anxiety and depression in ICU and elective cardiac surgery patients and their relatives. Intensive Care Med.

[43] Foster M, Chaboyer W (2003). Family carers of ICU survivors: a survey of the burden they experience. Scand J Caring Sci.

[44] Dithole K, Thupayagale-Tshweneagae G, Mgutshini T (2013). Posttraumatic stress disorder among spouses of patients discharged from the intensive care unit after six months. Issues Ment Health Nurs.

[45] Kim Y, Carver CS, Rocha-Lima C, Shaffer KM (2013). Depressive symptoms among caregivers of colorectal cancer patients during the first year since diagnosis: a longitudinal investigation. Psychooncology.

[46] Nieboer AP, Schulz R, Matthews KA, Scheier MF, Ormel J, Lindenberg SM (1998). Spousal caregivers’ activity restriction and depression: a model for changes over time. Soc Sci Med.

[47] Cooper C, Balamurali TB, Livingston G (2007). A systematic review of the prevalence and covariates of anxiety in caregivers of people with dementia. International Psychogeriatrics/IPA.

[48] Zigmond AS, Snaith RP (1983). The hospital anxiety and depression scale. Acta Psychiatr Scand.

[49] American Psychiatric Association (1994). Diagnostic and statistical manual of mental disorders: DSM-IV.

[50] Jones C, Skirrow P, Griffiths RD, Humphris GH, Ingleby S, Eddleston J (2003). Rehabilitation after critical illness: a randomized, controlled trial. Crit Care Med.

[51] Van der Schaaf M, Bakhshi-Raiez F, Van der Steen M, Dongelmans DA, De Keizer NF (2014). Recommendations for the organization of intensive care follow-up clinics; report from a survey and conference of Dutch intensive cares. Minerva Anestesiol.

