# Supplementary material for: Reported burden on informal caregivers of ICU survivors: a literature review
Source: Crit Care. 2016 Jan 21;20:16. doi: 10.1186/s13054-016-1185-9 (PMC4721206; doi:10.1186/s13054-016-1185-9)
Supplement: Supplementary file 1 — Informal caregiver characteristics. (DOC 206 kb) [file 13054_2016_1185_MOESM1_ESM.doc]

| **Additional table 1.** Characteristics of informal caregivers | | | | | | | | | | | |
| --- | --- | --- | --- | --- | --- | --- | --- | --- | --- | --- | --- |
| Author, year | Location | Design | n | Subgroup | Age  Mean±SD | Female (%) | Relationship to ICU patient (%) | Education  (%) | Employment  (%) | Ethnicity  (%) | Key inclusion ICU patient |
| Ågård, 2014 [1] | Denmark | Longitudinal observational descriptive | 18 |  | 57 (30-73)*^a^* | 61.1% | Partner 100% | - | Full-time 55.6%  Part-time 16.7%  Unemployed 5.6%  Retired 22.2% | - | ICU survivor  Age 25-70 year  intubated > 96 h |
| Anderson, 2008 [2] | USA | Prospective  longitudinal cohort | 50 |  | 54 (26-76)*^b^* | 84% | Spouse 36%  Parent 26%  Child 12%  Sibling/niece/nephew 6%  Unknown 20% | Attended college 34% | - | White 84%  African American 16% | ICU stay > 2 days |
| Azoulay, 2005 [3] | France | Longitudinal | 284 |  | 51 (41-61)*^c^* | 67.6% | Spouse 48.2%  Children 13.2%  Parents 23.9%  Other family 14.6% | - | Unemployed 28.3% | Not of European descent 13% | ICU stay > 2 days |
| Bayen, 2013 [4] | France | Prospective  inception  cohort | 66*^d^* |  | 50.3±13.1 | 73% | Parent 48%  Spouse 38%  Brother/sister 3%  Other family 8%  Other 3% | - | - | - | Age > 15 year,  severe TBI (i.e. GCS < 8 before hospital admission) |
| Cameron, 2006 [5] | Canada | Cross-sectional | 47 |  | 52.9±13.6 | 68.1% | Spouse 66.0%  Parent 23.4%  Other 6.4% | ≤ college 55.3%  ≥ university 36.2% | Working 40.4%  Caregiver/Home-maker 36.2%  Other 21.3% | - | Age ≥ 16 years,  ARDS survivors |
| Cox, 2009 [6] | USA | Semi-structured interviews | 24 |  | 53 (38-64)*^c^* | 83% | Spouse or partner 63%  Child 8%  Other family 25%  Friend 4% | - | - | White 79%  African-American 17%  Native American 4% | Required MV,  diagnosed with ARDS |
| Choi, 2011 [7] | USA | Longitudinal | 69*^e^* |  | < 30 year 23%  31-50 year 32%  51-70 year 46%  >70 year 13%  Unknown 6% | 52% | Spouse 55%  Adult child 22%  Parent/guardian 9%  Sibling 6%  Other 9% | - | Employed 59%  Unemployed/  retired 36%  Unknown 4% | White 91%  African- American 9% | Age ≥ 18 years ,  MV > 7 consecutive days |
| Choi, 2012 [8] | USA | Longitudinal descriptive | 50 |  | 52.3±11.8 | 74.0% | Spouse 58.0%  Adult child 24.0%  Parent/sibling 17.9% | Years of education  14.5±3.3 | Employed 54.0% | White 92.0% | Age ≥ 21 years,  MV ≥ 4 consecutive days |
| De Miranda, 2011 [9] | France | Prospective study | 102 |  | - | - | Spouse 53.9% | - | - | - | History of COPD,  ICU stay > 24 hrs for COPD exacerbation |
| Dithole, 2013 [10] | Botswana | - | 28 |  | 35.0 (26-54)*^c^* | 60.7% | Spouse 100% | None 14.3%  Primary 28.6%  HS 21.4%  Tertiary 35.7% | Working/Student 71.4%  No formal employment 28.6% | - | Received MV |
| Douglas, 2003 [11] | USA | Prospective longitudinal descriptive | 135 |  | 54.1±15.3 | 73.3% | Spouse 43.7%  Son/daughter 30.4%  Sibling 8.9%  Other relative 8.1%  Other 8.9% | - | Full-time 54.8%  Part-time 8.1%  Retired 18.5%  Not working/  not retired 18.5% | White 74.1% | Age ≥ 18 year,  MV > 4 days |
| Douglas, 2005 [12] | USA | Prospective experimental | 211 | Experimental | 53.1±14.5 | 73.9% | Spouse 39.8%  Son/daughter 31.3%  Sibling 9.0%  Other relative 14.7%  Other 5.2% | - | Employed 53.5%  Retired 25.0%  Not employed 21.5% | White 65.9% | Age ≥ 18 year  MV > 72 hours |
|  |  |  | 79 | Control | 52.6±17.7 | 68.4% | Spouse 46.8%  Son/daughter 34.2%  Sibling 12.7%  Other relative 5.1%  Other 1.3% | - | Employed 43.4%  Retired 27.6%  Not employed 28.9% | White 63.3% |  |
| Douglas, 2010 [13] | USA | Prospective study | 252 | White | 49.4±14.5 | 78.0% | Spouse 24.6%  Son/daughter 34.7%  Sibling 11.9%  Parent 27.1%  Other 1.7% | < HS 6.1%  ≥ HS < college 75.6%  ≥ college 18.2% | Employed 59.8% | White 100% | MV > 72 hours,  GCS < 6 |
|  |  |  | 118 | Non-white | 54.3±14.3 | 67.0% | Spouse 44.4%  Son/daughter 23.0%  Sibling 8.3%  Parent 18.7%  Other 5.6% | < HS 8.4%  ≥HS < college 65.9%  ≥ college 24.7% | Employed 58.7% | African-American 82.2%  Hispanic 10.2%  Asian 4.2%  Other 3.4% |  |
| Foster, 2003 [14] | Australia | Descriptive and correlational | 71 |  | 51.8±14.3 | 71.8% | Spouse 62.0%  Parent 11.3%  Adult child 23.9%  Other 2.8% | - | Full time 16.9%  Part time 8.5%  Casual 9.9%  Unpaid 38%  Unemployed 26.8% | - | ICU stay ≥ 5 days, admitted with a neurological condition |
| Garrouste-Orgeas, 2012 [15] | France | Prospective  single-center study | 48 | Pre-diary | - | - | Spouses 27.0%  Grown children 33.3%  Siblings 18.7%  Parents 12.5%  Other family 2.0%  Friends 6.2% | - | - | France 81.2%  Africa 10.4%  European countries ≠ France 4.1%  Other 4.1% | ICU stay ≥ 4 days |
|  |  |  | 49 | Diary | - | - | Spouses 44.9%  Grown children 28.5%  Siblings 8.1%  Parents 8.1%  Other family 10.2%  Friends 0% | - | - | France 83.6%  Africa 4.0%  European countries ≠ France 10.2%  Other 2.0% |  |
|  |  |  | 46 | Post-diary | - | - | Spouses 39.1%  Grown children 15.2%  Siblings 15.2%  Parents 21.7%  Other family 8.7%  Friends 0% | - | - | France 69.5%  Africa 17.3%  European countries ≠ France 2.1%  Other 10.8% |  |
| Im, 2004 [16] | USA | Prospective cohort | 115 |  | 52.9±14.2 | 76.5% | Spouse 52.2%  Adult child 18.3%  Parent/guardian 19.1%  Friend 0.9%  Others 9.6% | ≤ HS 55.7%  > HS 44.3% | Employed 28.6%  Homemaker 28.6%  Retired 21.7%  Other 20.8% | White 90.4%  Black 8.7%  Other 0.9% | Age ≥ 18 year  MV > 48 h |
| Jones, 2004 [17] | UK | RCT | 58 | Rehabilitation | 62±17 | 63% | Spouse/partner 50.0%  Adult child 20.7%  Parent 17.2%  Sibling 6.9%  Grandchild/niece 5.2% | - | - | British 100% | ICU stay >48 h, emergency admission and had been ventilated. |
|  |  |  | 46 | Control | 60±15.4 | 55% | Spouse/partner 54.3%  Adult child 17.4%  Parent 19.6%  Sibling 6.5%  Grandchild/niece 2.2% | - | - | British 100% |  |
| Jones, 2012 [18] | Sweden and UK | RCT | 15 | Intervention | - | 73.3% | Spouse/partner 56.7%  Child/child-in-law 26.7%  parent 10%  Siblings 6.7% | - | - | - | ICU stay ≥ 72 h and received MV ≥ 24 h |
|  |  |  | 15 | Control | - | 80.0% |  | - | - | - |  |
| Lemiale, 2010 [19] | France | Longitudinal  Observational | 284 |  | 51 (41–61)*^c^* | 67.6% | Spouse 48.2% | - | - | - | ICU stay of >48 h |
| McAdam, 2012 [20] | USA | Longitudinal descriptive | 74 *^f^* | - | 51.3±13.1 | 58.1% | Spouse/partner 43.2%  Adult child 33.8%  Parent 10.8%  Sibling 10.8%  Other 1.4% | ≥ college 71.6% | - | White 59.5%  Asian/Pacific Islander 20.3%  Hispanic 13.5%  Black 6.8% | Age ≥ 18 year  ICU stay ≥ 72 h  received MV, and an APACHE II ≥ 20 in the first 24 hours |
| Myhren, 2004 [21] | Norway | Prospective study | 50 |  | - | 32% | Spouses/cohabitant 40%  Child 30%  Parents 14%  Siblings 6%  Other 10% | - | - | - | Age ≥ 18 year  ICU stay ≥ 6 days  Receiving MV  Trachea intubated or tracheotomy |
| Rodríguez, 2005 [22] | Spain | - | 57 |  | 40.47*^g^* | 47.4% | Parents 49.1% | - | - | - | Age ≥ 14 year  Survived ICU admission |
| Rodríguez, 2005 [23] | Spain | - | 57 |  | 40.47*^g^* | 47.4% | Parents 49.1%  Husband/wife 19.3%  Brother/sister 17.5%  Son/daughter 8.8%  Others 5.3% | Completed primary studies 54.4% | - | - | head and brain trauma, poly-traumatized or traumatic quadri-plegics as an result of an unexpected accident |
| Swoboda, 2002 [24] | USA | - | 102 |  | - | 52% | Husband 22.7%  Wife 35.6%  Child 19.8%  Sibling 5%  Significant other 6% | - | Employed 74% | - | Admitted to a surgical ICU  ICU stay > 6 days |
| Van Pelt, 2007 [25] | USA | Prospective  parallel cohort | 169 |  | 54.6±14.7 | 75.7% | Spouse 52.7%  Other family 35.5%  Not family 11.8% | ≥ 12^th^ grade 88.8% | Employed 28.7% | White 91.1%  Black 8.3%  Other 0.6% | Age ≥ 18 year  Received MV ≥ 48 h |
| Van Pelt, 2010 [26] | USA | prospective, longitudinal observational | 48*^e^* |  | 52.8±12.8 | 81.2% | Spouse 47.9%  Other family 37.5%  Not family 14.6% | ≥ 12^th^ grade 87.5% | - | White 91.7%  Black 8.3% | Age ≥ 18 year  Received MV ≥ 48 h  Survived ≤ 12 months after initiation MV |
| Wartella, 2009 [27] | USA | - | 51 |  | 43.7 (19-84)*^b^* | 66% | - | - | - | Caucasian 52%  African American  42% | Diagnosed with TBI in the emergency department admitted to a neuroscience ICU |
| Young, 2005 [28] | UK | Single measure-ment point | 20 |  | 53.30±13.94 | 75% | - | - | - | - | Age ≥ 18 year  ICU stay ≥ 24 |

| *^a^* Median (range)  *^b^* Mean (range)  *^c^* Median (IQR)  *^d^* n baseline measured during (first) follow-up period  *^e^* n informal caregivers who completed all follow-up points  *^f^* n baseline measured during ICU stay  *^g^* Mean | TBI: Traumatic brain injury  ICU: Intensive care unit  COPD: Chronic obstructive pulmonary disease  USA: United States of America  UK: United Kingdom  RCT: Randomised controlled trial | MV: Mechanical ventilation  GCS: Glasgow Coma Scale  ARDS: Acute respiratory distress syndrome  HS: High school  APACHE II: Acute Physiology and Chronic Health Evaluation II |
| --- | --- | --- |

1. Agard AS, Lomborg K, Tonnesen E, Egerod I. Rehabilitation activities, out-patient visits and employment in patients and partners the first year after ICU: a descriptive study. Intensive & critical care nursing : the official journal of the British Association of Critical Care Nurses. 2014;30(2):101-10. doi:10.1016/j.iccn.2013.11.001.

2. Anderson WG, Arnold RM, Angus DC, Bryce CL. Posttraumatic stress and complicated grief in family members of patients in the intensive care unit. Journal of general internal medicine. 2008;23(11):1871-6. doi:10.1007/s11606-008-0770-2.

3. Azoulay E, Pochard F, Kentish-Barnes N, Chevret S, Aboab J, Adrie C et al. Risk of post-traumatic stress symptoms in family members of intensive care unit patients. American journal of respiratory and critical care medicine. 2005;171(9):987-94. doi:10.1164/rccm.200409-1295OC.

4. Bayen E, Pradat-Diehl P, Jourdan C, Ghout I, Bosserelle V, Azerad S et al. Predictors of informal care burden 1 year after a severe traumatic brain injury: results from the PariS-TBI study. The Journal of head trauma rehabilitation. 2013;28(6):408-18. doi:10.1097/HTR.0b013e31825413cf.

5. Cameron JI, Herridge MS, Tansey CM, McAndrews MP, Cheung AM. Well-being in informal caregivers of survivors of acute respiratory distress syndrome. Critical care medicine. 2006;34(1):81-6.

6. Cox CE, Docherty SL, Brandon DH, Whaley C, Attix DK, Clay AS et al. Surviving critical illness: acute respiratory distress syndrome as experienced by patients and their caregivers. Critical care medicine. 2009;37(10):2702-8. doi:10.1097/CCM.0b013e3181b6f64a.

7. Choi J, Donahoe MP, Zullo TG, Hoffman LA. Caregivers of the chronically critically ill after discharge from the intensive care unit: six months' experience. American journal of critical care : an official publication, American Association of Critical-Care Nurses. 2011;20(1):12-22; quiz 3. doi:10.4037/ajcc2011243.

8. Choi J, Sherwood PR, Schulz R, Ren D, Donahoe MP, Given B et al. Patterns of depressive symptoms in caregivers of mechanically ventilated critically ill adults from intensive care unit admission to 2 months postintensive care unit discharge: a pilot study. Critical care medicine. 2012;40(5):1546-53. doi:10.1097/CCM.0b013e3182451c58.

9. de Miranda S, Pochard F, Chaize M, Megarbane B, Cuvelier A, Bele N et al. Postintensive care unit psychological burden in patients with chronic obstructive pulmonary disease and informal caregivers: A multicenter study. Critical care medicine. 2011;39(1):112-8. doi:10.1097/CCM.0b013e3181feb824.

10. Dithole K, Thupayagale-Tshweneagae G, Mgutshini T. Posttraumatic stress disorder among spouses of patients discharged from the intensive care unit after six months. Issues in mental health nursing. 2013;34(1):30-5. doi:10.3109/01612840.2012.715235.

11. Douglas SL, Daly BJ. Caregivers of long-term ventilator patients: physical and psychological outcomes. Chest. 2003;123(4):1073-81.

12. Douglas SL, Daly BJ, Kelley CG, O'Toole E, Montenegro H. Impact of a disease management program upon caregivers of chronically critically ill patients. Chest. 2005;128(6):3925-36. doi:10.1378/chest.128.6.3925.

13. Douglas SL, Daly BJ, O'Toole E, Hickman RL, Jr. Depression among white and nonwhite caregivers of the chronically critically ill. Journal of critical care. 2010;25(2):364 e11-9. doi:10.1016/j.jcrc.2009.09.004.

14. Foster M, Chaboyer W. Family carers of ICU survivors: a survey of the burden they experience. Scandinavian journal of caring sciences. 2003;17(3):205-14.

15. Garrouste-Orgeas M, Coquet I, Perier A, Timsit JF, Pochard F, Lancrin F et al. Impact of an intensive care unit diary on psychological distress in patients and relatives*. Critical care medicine. 2012;40(7):2033-40. doi:10.1097/CCM.0b013e31824e1b43.

16. Im K, Belle SH, Schulz R, Mendelsohn AB, Chelluri L, Investigators Q-M. Prevalence and outcomes of caregiving after prolonged (> or =48 hours) mechanical ventilation in the ICU. Chest. 2004;125(2):597-606.

17. Jones C, Skirrow P, Griffiths RD, Humphris G, Ingleby S, Eddleston J et al. Post-traumatic stress disorder-related symptoms in relatives of patients following intensive care. Intensive care medicine. 2004;30(3):456-60. doi:10.1007/s00134-003-2149-5.

18. Jones C, Backman C, Griffiths RD. Intensive care diaries and relatives' symptoms of posttraumatic stress disorder after critical illness: a pilot study. American journal of critical care : an official publication, American Association of Critical-Care Nurses. 2012;21(3):172-6. doi:10.4037/ajcc2012569.

19. Lemiale V, Kentish-Barnes N, Chaize M, Aboab J, Adrie C, Annane D et al. Health-related quality of life in family members of intensive care unit patients. Journal of palliative medicine. 2010;13(9):1131-7. doi:10.1089/jpm.2010.0109.

20. McAdam JL, Fontaine DK, White DB, Dracup KA, Puntillo KA. Psychological symptoms of family members of high-risk intensive care unit patients. American journal of critical care : an official publication, American Association of Critical-Care Nurses. 2012;21(6):386-93; quiz 94. doi:10.4037/ajcc2012582.

21. Myhren H, Ekeberg O, Langen I, Stokland O. Emotional strain, communication, and satisfaction of family members in the intensive care unit compared with expectations of the medical staff: experiences from a Norwegian University Hospital. Intensive care medicine. 2004;30(9):1791-8. doi:10.1007/s00134-004-2375-5.

22. Rodríguez AM, Gregorio MA, Rodriguez AG. Psychological repercussions in family members of hospitalised critical condition patients. Journal of psychosomatic research. 2005;58(5):447-51. doi:10.1016/j.jpsychores.2004.11.011.

23. Rodríguez AM. Psychosocial adaptation in relatives of critically injured patients admitted to an intensive care unit. The Spanish Journal of Psychology. 2005;8(1, 36-44):1138-7416.

24. Swoboda SM, Lipsett PA. Impact of a prolonged surgical critical illness on patients' families. American journal of critical care : an official publication, American Association of Critical-Care Nurses. 2002;11(5):459-66.

25. Van Pelt DC, Milbrandt EB, Qin L, Weissfeld LA, Rotondi AJ, Schulz R et al. Informal caregiver burden among survivors of prolonged mechanical ventilation. American journal of respiratory and critical care medicine. 2007;175(2):167-73. doi:10.1164/rccm.200604-493OC.

26. Van Pelt DC, Schulz R, Chelluri L, Pinsky MR. Patient-specific, time-varying predictors of post-ICU informal caregiver burden: the caregiver outcomes after ICU discharge project. Chest. 2010;137(1):88-94. doi:10.1378/chest.09-0795.

27. Wartella JE, Auerbach SM, Ward KR. Emotional distress, coping and adjustment in family members of neuroscience intensive care unit patients. Journal of psychosomatic research. 2009;66(6):503-9. doi:10.1016/j.jpsychores.2008.12.005.

28. Young E, Eddleston J, Ingleby S, Streets J, McJanet L, Wang M et al. Returning home after intensive care: a comparison of symptoms of anxiety and depression in ICU and elective cardiac surgery patients and their relatives. Intensive care medicine. 2005;31(1):86-91. doi:10.1007/s00134-004-2495-y.
