# Supplementary material for: Reported burden on informal caregivers of ICU survivors: a literature review
Source: Crit Care. 2016 Jan 21;20:16. doi: 10.1186/s13054-016-1185-9 (PMC4721206; doi:10.1186/s13054-016-1185-9)
Supplement: Supplementary file 2 — Overview of all types of burden reported in the included articles. (DOC 528 kb) [file 13054_2016_1185_MOESM2_ESM.doc]

| **Additional Table 2.** Overview of all burden reported in the included articles | |
| --- | --- |
| **Themes** | **Burden** |
| Psychological | PTSD [1-8]  Anxiety [1, 2, 6, 7, 3, 9-11, 5, 8, 12]  Depression [1, 2, 13-16, 7, 17, 3, 18-20, 9-11, 5, 8, 12, 6]  Suicidal depression [21, 22], anxious depression [21, 22]  Emotional distress [23, 9], emotional problems [14], emotional instability [6], emotional burden [24]  Distress [25, 26]  Feeling overloaded as a result of caregiving activities [15]  Hopelessness [26]  Overwhelmed [26, 18]  Insomnia [12], sleep disorders [5], sleep disturbances [6], poor sleep patterns, including nightmares, waking up at odd hours, and struggling to fall asleep [6], “I would keep myself awake to check he was still breathing”[10], restriction in sleeping habits [25]  Concentration problems [12, 6]  Intrusion [6-8]  Fear [6], Fear of down death [21, 22]  Crying for no apparent reason [6]  Startle reactions [6]  Feeling cut off from people [6]  Re-experiencing the event [6], Avoidance [6-8]  Hyper arousal symptoms [6-8]  Peritraumatic dissociation [7]  Sadness [5]  Somatization [9]  Hostility [9], Agitation [21, 22]  Denial [9]  Hypochondriasis [21, 22]  Low Energy Level [21, 22]  Guilt-resentment [21, 22]  Boredom-withdrawal [21, 22]  Paranoia [21, 22]  Psychopathic deviation [21, 22]  Schizophrenia [21, 22]  Psychasthenia [21, 22]  Psychological Inadequacy [21, 22] |
| Physical | Physical problems such as pain and arthritis [27]  Poor physical health [15], physical burden [24]  Exhaustion [26], having little less energy [15]  Physical reactions such as sweating or palpations [6]  Health problems [18] |
| HRQoL | Decreased quality of life [2], decline in self-perceived quality of life [5]  Poor health status [13],decrease of overall health status [16]  Low HRQoL [23], alterations in HRQoL [11] |
| Social | Complicated logistics of home life [27]  Social problems [27], social burden [24]  “Now we are in all the time” [10], “not able to go out as much as before” [10], “I have lost interest in everything. I do not go to the saloon, do not visit my friends. I am afraid of life” [6]  Lack of support [26, 18]  Strain of balancing child care and work [26]  Keeping distant from family and friends [6], generalised dissociation from people [6]  Restrictions in visiting friends, hobbies, sport and recreation, shopping for self, doing household chores, caring for self, caring for others, eating habits, and maintaining friendship [25] |
| Relationship | Separation [27]  “Freedom in relationship has lessened” [10]  “More irritable with each other” [10]  increased distance in relationship [26]  “I do not think we have a real normal marriage now”[26]  Feeling of irritation with the ICU patient [26]  Feeling of anger with the ICU patient [26]  “It was hard living with him [the ICU patient]” [26]  “Our relationship will never be the same. It is all gone . . . different” [26]  “we are sinking” [26] |
| Financial | Difficulties to pay for basics such as food, housing, medical care, and heating [17]  Most of savings lost [28]  Major source of family income lost [28]  Moved to a less expensive home because of the cost of the illness [28]  Delayed medical care for themselves because of the cost of the illness [28]  Altered educational plans because of the cost of the illness [28]  Financial pressures to return to work [26]  Having little less money [15] |
| Employment | Los of employment [27]  Reduction in employment [19], restrictions in work [25]  Early retirement to become an informal caregiver [27]  Sick leave from work [27]  Often taking a day off from work to drive the patient to clinic visits [27]  Reduced work hours for caregiving role [16, 17]  Quit work for caregiving role [16, 28, 17]  Fired as a result of the caregiving role [16]  “When I came back, I did not have that [multimillion-dollar] project anymore. They seem to have forgotten they promoted me” [26] |
| Other | Long commutes to rehabilitation facility [27]  Burden [13, 18], Caregiver burden [14]  Lifestyle interference [23], lifestyle restriction [19], lifestyle disruption [20], restriction of activities [17], disrupted schedule [18]  Lower levels of mastery [23]  Having little less time [15], time-dependent burden [24]  Having little less privacy [15], having little less personal freedom [15]  Developmental burden [24]  “Did not have any back up at home” [10]  A lingering feeling of regret [26]  “Doing a lot more jobs now.”[10] |
| Spiritual | Fear related to supernatural phenomena [6]  Fear that ICU patient had been “bewitched” [6] |

1. Anderson WG, Arnold RM, Angus DC, Bryce CL. Posttraumatic stress and complicated grief in family members of patients in the intensive care unit. Journal of general internal medicine. 2008;23(11):1871-6. doi:10.1007/s11606-008-0770-2.

2. Azoulay E, Pochard F, Kentish-Barnes N, Chevret S, Aboab J, Adrie C et al. Risk of post-traumatic stress symptoms in family members of intensive care unit patients. American journal of respiratory and critical care medicine. 2005;171(9):987-94. doi:10.1164/rccm.200409-1295OC.

3. Jones C, Skirrow P, Griffiths RD, Humphris G, Ingleby S, Eddleston J et al. Post-traumatic stress disorder-related symptoms in relatives of patients following intensive care. Intensive care medicine. 2004;30(3):456-60. doi:10.1007/s00134-003-2149-5.

4. Jones C, Backman C, Griffiths RD. Intensive care diaries and relatives' symptoms of posttraumatic stress disorder after critical illness: a pilot study. American journal of critical care : an official publication, American Association of Critical-Care Nurses. 2012;21(3):172-6. doi:10.4037/ajcc2012569.

5. de Miranda S, Pochard F, Chaize M, Megarbane B, Cuvelier A, Bele N et al. Postintensive care unit psychological burden in patients with chronic obstructive pulmonary disease and informal caregivers: A multicenter study. Critical care medicine. 2011;39(1):112-8. doi:10.1097/CCM.0b013e3181feb824.

6. Dithole K, Thupayagale-Tshweneagae G, Mgutshini T. Posttraumatic stress disorder among spouses of patients discharged from the intensive care unit after six months. Issues in mental health nursing. 2013;34(1):30-5. doi:10.3109/01612840.2012.715235.

7. Garrouste-Orgeas M, Coquet I, Perier A, Timsit JF, Pochard F, Lancrin F et al. Impact of an intensive care unit diary on psychological distress in patients and relatives*. Critical care medicine. 2012;40(7):2033-40. doi:10.1097/CCM.0b013e31824e1b43.

8. McAdam JL, Fontaine DK, White DB, Dracup KA, Puntillo KA. Psychological symptoms of family members of high-risk intensive care unit patients. American journal of critical care : an official publication, American Association of Critical-Care Nurses. 2012;21(6):386-93; quiz 94. doi:10.4037/ajcc2012582.

9. Wartella JE, Auerbach SM, Ward KR. Emotional distress, coping and adjustment in family members of neuroscience intensive care unit patients. Journal of psychosomatic research. 2009;66(6):503-9. doi:10.1016/j.jpsychores.2008.12.005.

10. Young E, Eddleston J, Ingleby S, Streets J, McJanet L, Wang M et al. Returning home after intensive care: a comparison of symptoms of anxiety and depression in ICU and elective cardiac surgery patients and their relatives. Intensive care medicine. 2005;31(1):86-91. doi:10.1007/s00134-004-2495-y.

11. Lemiale V, Kentish-Barnes N, Chaize M, Aboab J, Adrie C, Annane D et al. Health-related quality of life in family members of intensive care unit patients. Journal of palliative medicine. 2010;13(9):1131-7. doi:10.1089/jpm.2010.0109.

12. Myhren H, Ekeberg O, Langen I, Stokland O. Emotional strain, communication, and satisfaction of family members in the intensive care unit compared with expectations of the medical staff: experiences from a Norwegian University Hospital. Intensive care medicine. 2004;30(9):1791-8. doi:10.1007/s00134-004-2375-5.

13. Bayen E, Pradat-Diehl P, Jourdan C, Ghout I, Bosserelle V, Azerad S et al. Predictors of informal care burden 1 year after a severe traumatic brain injury: results from the PariS-TBI study. The Journal of head trauma rehabilitation. 2013;28(6):408-18. doi:10.1097/HTR.0b013e31825413cf.

14. Choi J, Sherwood PR, Schulz R, Ren D, Donahoe MP, Given B et al. Patterns of depressive symptoms in caregivers of mechanically ventilated critically ill adults from intensive care unit admission to 2 months postintensive care unit discharge: a pilot study. Critical care medicine. 2012;40(5):1546-53. doi:10.1097/CCM.0b013e3182451c58.

15. Douglas SL, Daly BJ. Caregivers of long-term ventilator patients: physical and psychological outcomes. Chest. 2003;123(4):1073-81.

16. Douglas SL, Daly BJ, O'Toole E, Hickman RL, Jr. Depression among white and nonwhite caregivers of the chronically critically ill. Journal of critical care. 2010;25(2):364 e11-9. doi:10.1016/j.jcrc.2009.09.004.

17. Im K, Belle SH, Schulz R, Mendelsohn AB, Chelluri L, Investigators Q-M. Prevalence and outcomes of caregiving after prolonged (> or =48 hours) mechanical ventilation in the ICU. Chest. 2004;125(2):597-606.

18. Douglas SL, Daly BJ, Kelley CG, O'Toole E, Montenegro H. Impact of a disease management program upon caregivers of chronically critically ill patients. Chest. 2005;128(6):3925-36. doi:10.1378/chest.128.6.3925.

19. Van Pelt DC, Milbrandt EB, Qin L, Weissfeld LA, Rotondi AJ, Schulz R et al. Informal caregiver burden among survivors of prolonged mechanical ventilation. American journal of respiratory and critical care medicine. 2007;175(2):167-73. doi:10.1164/rccm.200604-493OC.

20. Van Pelt DC, Schulz R, Chelluri L, Pinsky MR. Patient-specific, time-varying predictors of post-ICU informal caregiver burden: the caregiver outcomes after ICU discharge project. Chest. 2010;137(1):88-94. doi:10.1378/chest.09-0795.

21. Rodríguez AM. Psychosocial adaptation in relatives of critically injured patients admitted to an intensive care unit. The Spanish Journal of Psychology. 2005;8(1, 36-44):1138-7416.

22. Rodríguez AM, Gregorio MA, Rodriguez AG. Psychological repercussions in family members of hospitalised critical condition patients. Journal of psychosomatic research. 2005;58(5):447-51. doi:10.1016/j.jpsychores.2004.11.011.

23. Cameron JI, Herridge MS, Tansey CM, McAndrews MP, Cheung AM. Well-being in informal caregivers of survivors of acute respiratory distress syndrome. Critical care medicine. 2006;34(1):81-6.

24. Foster M, Chaboyer W. Family carers of ICU survivors: a survey of the burden they experience. Scandinavian journal of caring sciences. 2003;17(3):205-14.

25. Choi J, Donahoe MP, Zullo TG, Hoffman LA. Caregivers of the chronically critically ill after discharge from the intensive care unit: six months' experience. American journal of critical care : an official publication, American Association of Critical-Care Nurses. 2011;20(1):12-22; quiz 3. doi:10.4037/ajcc2011243.

26. Cox CE, Docherty SL, Brandon DH, Whaley C, Attix DK, Clay AS et al. Surviving critical illness: acute respiratory distress syndrome as experienced by patients and their caregivers. Critical care medicine. 2009;37(10):2702-8. doi:10.1097/CCM.0b013e3181b6f64a.

27. Agard AS, Lomborg K, Tonnesen E, Egerod I. Rehabilitation activities, out-patient visits and employment in patients and partners the first year after ICU: a descriptive study. Intensive & critical care nursing : the official journal of the British Association of Critical Care Nurses. 2014;30(2):101-10. doi:10.1016/j.iccn.2013.11.001.

28. Swoboda SM, Lipsett PA. Impact of a prolonged surgical critical illness on patients' families. American journal of critical care : an official publication, American Association of Critical-Care Nurses. 2002;11(5):459-66.
