# Supplementary material for: Reported burden on informal caregivers of ICU survivors: a literature review
Source: Crit Care. 2016 Jan 21;20:16. doi: 10.1186/s13054-016-1185-9 (PMC4721206; doi:10.1186/s13054-016-1185-9)
Supplement: Supplementary file 3 — Depression: assessment tools, time points and outcomes measures for caregivers for quantitative studies. (DOC 179 kb) [file 13054_2016_1185_MOESM3_ESM.doc]

| **Additional table 3.** Depression: Assessment tools, time points and outcomes measures for caregivers for quantitative studies | | | | | | | | |
| --- | --- | --- | --- | --- | --- | --- | --- | --- |
| Author, year | Assessment tool | Score range | Cut-off score | n | Subgroup | Time of measurement | Prevalence % | Mean±SD |
| Anderson, 2008 [1] | HADS | 0-21 | ≥ 11 | 50 | - | During ICU stay | 16% | 7±4 |
|  |  |  |  | 39 |  | 1 month after enrolment | 8% | 4±4 |
|  |  |  |  | 34 |  | 6 months after enrolment | 6% | 4±3 |
| Azoulay, 2005 [2] | HADS | 0-21 | ≥ 11 | 284 | - | 3 months after ICU discharge or death | 20.1% | - |
| Bayen, 2013 [3] | ZBI | 0-88 | > 24 | 66 | - | 1 year after injury | 44% | 25.1±17.2 |
| Cameron, 2006 [4] | CES-D | 0-60 | ≥ 16 | 47 | - | On average 23 months after hospital discharge | 31.9% | 12 (4-23)*^a^* |
| Choi, 2012 [5] | shortened CES-D | 0-30 | ≥8 | 50 | - | During ICU admission | 90% | 16.4±7.1 |
|  |  |  |  | 41 | - | At ICU discharge | 73% | 10.5±5.9 |
|  |  |  |  | 31 | - | 2 months after ICU discharge. | 61% | 10.3±5.9 |
| de Miranda, 2011 [6] | HADS | 0-21 | ≥ 8 | 102 | - | At ICU discharge | 25.7% | - |
|  |  |  |  | 47 |  | 3 months after ICU discharge | 14.9% | - |
| Douglas, 2003 [7] | CES-D | 0-60 | ≥ 16 | 135 | - | At hospital discharge | 51.2% | 15.5±11.8 |
|  |  |  |  | 77 | - | 6 months after hospital discharge | 36.4% | 13.9±12.8 |
| Douglas, 2005 [8] | CES-D | 0-60 | ≥ 16 | 206 | Experimental | At hospital discharge | 52.9% | 17.8±12.3 |
|  |  |  |  | 79 | Control |  | 41.0% | 15.8±10.9 |
|  |  |  |  | 166 | Experimental | 2 months after hospital discharge | 31.3% | 12.3±11.5 |
|  |  |  |  | 56 | Control |  | 29.2% | 12.2±11.4 |
| Douglas, 2010 [9] | CES-D | 0-60 | ≥16 | 252 | white | ICU admission | 75.2% | 23.7±10.9 |
|  |  |  |  | 118 | Non-white |  | 72.0% | 23.4±11.2 |
|  |  |  |  | 193 | White | 2 months after hospital discharge | 45.4% | 15.7±12.8 |
|  |  |  |  | 84 | Non-white |  | 50.5% | 16.8±13.2 |
| Garrouste-Orgeas, 2012 [10] | HADS | 0-21 | ≥8 | 48 | Pre-diary | ICU discharge | 25% | 5.1±4.1 |
|  |  |  |  | 49 | Diary |  | 30.6% | 6.6±4.6 |
|  |  |  |  | 46 | Post-diary |  | 41.3% | 7.5±3.9 |
|  |  |  |  | 48 | Pre-diary | 3 months after ICU discharge | 18.8% | 4.7±5.0 |
|  |  |  |  | 46 | Diary |  | 21.7% | 4.1±4.4 |
|  |  |  |  | 42 | Post-diary |  | 26.2% | 5.1±4.5 |
| Im, 2004 [11] | CES-D | 0-60 | ≥ 16 | 115 | - | 2 months following the onset of prolonged MV | 33.9% | 13.2±11 |
| Jones, 2004 [12] | HADS | 0-21 | ≥ 11 | 58 | Rehabilitation | On the general ward | 22% | 6.5±4.38 |
|  |  |  |  | 46 | Control |  | 31% | 7±4.52 |
|  |  |  |  | 50 | Rehabilitation | 2 months after ICU discharge | 7.8% | 3.8±3.42 |
|  |  |  |  | 40 | Control |  | 7% | 4.6±4 |
|  |  |  |  | 47 | Rehabilitation | 6 months after ICU discharge | 4.7% | 3.7±3.51 |
|  |  |  |  | 37 | Control |  | 5.6% | 4.6±4.1 |
| Lemiale, 2010 [13] | HADS | 0-21 | - | 284 | - | 3 months ICU discharge or death | 20.1% | - |
| McAdam, 2012 [14] | HADS | 0-21 | ≥ 8 | 74 | - | During ICU stay | 70.3% | 9.62±4.2 |
|  |  |  | ≥ 11 |  |  |  | 43.2% |  |
|  |  |  | ≥ 8 | 41 | - | 3 months after ICU discharge or death | 26.8% | 5.6±4.6 |
|  |  |  | ≥ 11 |  |  |  | 12.2% |  |
| Myhren, 2004 [15] | Self-developed questionnaire | - | - | 50 | - | During ICU stay | - | - |
|  |  |  |  | 50 | - | 1 month after the ICU stay | - | - |
| Van Pelt, 2007 [16] | CES-D | 0-60 | ≥ 16 | 115 | - | 2 months after MV initiation | 33.9% | 13.2±11.0 |
|  |  |  |  | 107 | - | 6 months after MV initiation | 30.8% | 12.7±11.2 |
|  |  |  |  | 92 | - | 12 months after MV initiation | 22.8% | 10.6±10.7 |
| Van Pelt, 2010 [17] | CES-D | 0-60 | ≥ 16 | 48 | - | 2 months after MV initiation | 37.5% | 13.4±12.4 |
|  |  |  |  | 48 | - | 6 months after MV initiation | 29.2% | 12.3±12.0 |
|  |  |  |  | 48 | - | 12 months after MV initiation | 29.2% | 11.8±12.1 |
| Wartella, 2009 [18] | BSI - Depression | - | - | 51 | - | At ICU admission | - | 0.36±0.62 |
|  |  |  |  | 51 | - | At ICU discharge | - | 0.27±0.50 |
|  |  |  |  | 51 | - | 1 month after ICU discharge | - | 0.16±0.33 |
| Young, 2005 [19] | HADS | 0-21 | ≥ 8 | 20 | - | 3 months after ICU discharge | 25% | - |
|  |  |  | ≥ 11 |  |  |  | 15% | - |

| CES-D: Centre for Epidemiological Studies-Depression  HADS: Hospital Anxiety and Depression Scale  ICU: Intensive care unit  MV: Mechanical ventilation  ZBI: Zarit Burden Inventory  BSI: Brief Symptom Inventory | *^a^* Median (IQR) |
| --- | --- |

1. Anderson WG, Arnold RM, Angus DC, Bryce CL. Posttraumatic stress and complicated grief in family members of patients in the intensive care unit. Journal of general internal medicine. 2008;23(11):1871-6. doi:10.1007/s11606-008-0770-2.

2. Azoulay E, Pochard F, Kentish-Barnes N, Chevret S, Aboab J, Adrie C et al. Risk of post-traumatic stress symptoms in family members of intensive care unit patients. American journal of respiratory and critical care medicine. 2005;171(9):987-94. doi:10.1164/rccm.200409-1295OC.

3. Bayen E, Pradat-Diehl P, Jourdan C, Ghout I, Bosserelle V, Azerad S et al. Predictors of informal care burden 1 year after a severe traumatic brain injury: results from the PariS-TBI study. The Journal of head trauma rehabilitation. 2013;28(6):408-18. doi:10.1097/HTR.0b013e31825413cf.

4. Cameron JI, Herridge MS, Tansey CM, McAndrews MP, Cheung AM. Well-being in informal caregivers of survivors of acute respiratory distress syndrome. Critical care medicine. 2006;34(1):81-6.

5. Choi J, Sherwood PR, Schulz R, Ren D, Donahoe MP, Given B et al. Patterns of depressive symptoms in caregivers of mechanically ventilated critically ill adults from intensive care unit admission to 2 months postintensive care unit discharge: a pilot study. Critical care medicine. 2012;40(5):1546-53. doi:10.1097/CCM.0b013e3182451c58.

6. de Miranda S, Pochard F, Chaize M, Megarbane B, Cuvelier A, Bele N et al. Postintensive care unit psychological burden in patients with chronic obstructive pulmonary disease and informal caregivers: A multicenter study. Critical care medicine. 2011;39(1):112-8. doi:10.1097/CCM.0b013e3181feb824.

7. Douglas SL, Daly BJ. Caregivers of long-term ventilator patients: physical and psychological outcomes. Chest. 2003;123(4):1073-81.

8. Douglas SL, Daly BJ, Kelley CG, O'Toole E, Montenegro H. Impact of a disease management program upon caregivers of chronically critically ill patients. Chest. 2005;128(6):3925-36. doi:10.1378/chest.128.6.3925.

9. Douglas SL, Daly BJ, O'Toole E, Hickman RL, Jr. Depression among white and nonwhite caregivers of the chronically critically ill. Journal of critical care. 2010;25(2):364 e11-9. doi:10.1016/j.jcrc.2009.09.004.

10. Garrouste-Orgeas M, Coquet I, Perier A, Timsit JF, Pochard F, Lancrin F et al. Impact of an intensive care unit diary on psychological distress in patients and relatives*. Critical care medicine. 2012;40(7):2033-40. doi:10.1097/CCM.0b013e31824e1b43.

11. Im K, Belle SH, Schulz R, Mendelsohn AB, Chelluri L, Investigators Q-M. Prevalence and outcomes of caregiving after prolonged (> or =48 hours) mechanical ventilation in the ICU. Chest. 2004;125(2):597-606.

12. Jones C, Skirrow P, Griffiths RD, Humphris G, Ingleby S, Eddleston J et al. Post-traumatic stress disorder-related symptoms in relatives of patients following intensive care. Intensive care medicine. 2004;30(3):456-60. doi:10.1007/s00134-003-2149-5.

13. Lemiale V, Kentish-Barnes N, Chaize M, Aboab J, Adrie C, Annane D et al. Health-related quality of life in family members of intensive care unit patients. Journal of palliative medicine. 2010;13(9):1131-7. doi:10.1089/jpm.2010.0109.

14. McAdam JL, Fontaine DK, White DB, Dracup KA, Puntillo KA. Psychological symptoms of family members of high-risk intensive care unit patients. American journal of critical care : an official publication, American Association of Critical-Care Nurses. 2012;21(6):386-93; quiz 94. doi:10.4037/ajcc2012582.

15. Myhren H, Ekeberg O, Langen I, Stokland O. Emotional strain, communication, and satisfaction of family members in the intensive care unit compared with expectations of the medical staff: experiences from a Norwegian University Hospital. Intensive care medicine. 2004;30(9):1791-8. doi:10.1007/s00134-004-2375-5.

16. Van Pelt DC, Milbrandt EB, Qin L, Weissfeld LA, Rotondi AJ, Schulz R et al. Informal caregiver burden among survivors of prolonged mechanical ventilation. American journal of respiratory and critical care medicine. 2007;175(2):167-73. doi:10.1164/rccm.200604-493OC.

17. Van Pelt DC, Schulz R, Chelluri L, Pinsky MR. Patient-specific, time-varying predictors of post-ICU informal caregiver burden: the caregiver outcomes after ICU discharge project. Chest. 2010;137(1):88-94. doi:10.1378/chest.09-0795.

18. Wartella JE, Auerbach SM, Ward KR. Emotional distress, coping and adjustment in family members of neuroscience intensive care unit patients. Journal of psychosomatic research. 2009;66(6):503-9. doi:10.1016/j.jpsychores.2008.12.005.

19. Young E, Eddleston J, Ingleby S, Streets J, McJanet L, Wang M et al. Returning home after intensive care: a comparison of symptoms of anxiety and depression in ICU and elective cardiac surgery patients and their relatives. Intensive care medicine. 2005;31(1):86-91. doi:10.1007/s00134-004-2495-y.
