# Supplementary material for: Reported burden on informal caregivers of ICU survivors: a literature review
Source: Crit Care. 2016 Jan 21;20:16. doi: 10.1186/s13054-016-1185-9 (PMC4721206; doi:10.1186/s13054-016-1185-9)
Supplement: Supplementary file 4 — Anxiety: assessment tools, time points and outcomes measures for caregivers for quantitative studies. (DOC 93 kb) [file 13054_2016_1185_MOESM4_ESM.doc]

| **Supplemental table 4.** Anxiety: Assessment tools, time points and outcomes measures for caregivers for quantitative studies | | | | | | | | |
| --- | --- | --- | --- | --- | --- | --- | --- | --- |
| Author, year | Assessment tool | Score range | Cut-off score | n | Subgroup | Time of measurement | Prevalence % | Mean±SD |
| Anderson, 2008 [1] | HADS | 0-21 | ≥ 11 | 50 | - | During ICU stay | 42% | 10±4 |
|  |  |  |  | 39 | - | 1 month after enrolment | 21% | 7±5 |
|  |  |  |  | 34 | - | 6 months after enrolment | 15% | 6±5 |
| Azoulay, 2005 [2] | HADS | 0-21 | ≥ 11 | 284 | - | 3 months after ICU discharge or death | 49.3% | - |
| De Miranda, 2011 [3] | HADS | 0-21 | ≥ 8 | 102 | - | At ICU discharge | 72.2% | - |
|  |  |  |  | 47 | - | 3 months after ICU discharge | 40.4% | - |
| Garrouste-Orgeas, 2012 [4] | HADS | 0-21 | ≥ 8 | 48 | Pre-diary | At ICU discharge | 47.9% | 7.9±3.3 |
|  |  |  |  | 49 | Diary |  | 50.0% | 7.9±3.0 |
|  |  |  |  | 46 | Post-diary |  | 59.5% | 8.7±2.7 |
|  |  |  |  | 48 | Pre-diary | 3 months after ICU discharge | 62.5% | 10.4±4.8 |
|  |  |  |  | 46 | Diary |  | 39.1% | 7.6±4.8 |
|  |  |  |  | 42 | Post-diary |  | 61.9% | 8.8±4.3 |
| Jones, 2004 [5] | HADS | 0-21 | ≥ 11 | 58 | Rehabilitation | On the general ward | 58% | 11±4.85 |
|  |  |  |  | 46 | Control |  | 62% | 12±4.43 |
|  |  |  |  | 50 | Rehabilitation | 2 months after ICU discharge | 27% | 6.9±4.84 |
|  |  |  |  | 40 | Control |  | 35% | 7.8±4.71 |
|  |  |  |  | 47 | Rehabilitation | 6 months after ICU discharge | 22% | 6.8±5.03 |
|  |  |  |  | 37 | Control |  | 24% | 3.7±4.61 |
| Lemiale, 2010 [6] | HADS | 0-21 | - | 284 | - | 3 months after ICU discharge or death | 49.3% | - |
| McAdam, 2012 [7] | HADS | 0-21 | ≥ 8 | 74 | - | During ICU stay | 79.7% | 11.80±4.7 |
|  |  |  | ≥ 11 |  |  |  | 59.5% |  |
|  |  |  | ≥ 8 | 41 | - | 3 months after ICU discharge or death | 43.9% | 7.3±3.9 |
|  |  |  | ≥ 11 |  |  |  | 24.4% |  |
| Myhren, 2004 [8] | Self-developed questionnaire | - | - | 50 | - | During ICU stay | - | - |
|  |  |  |  | 50 | - | 1 month after the ICU stay | - | 3.0±1.0 |
| Wartella, 2009 [9] | BSI - Anxiety | - | - | 51 | - | At ICU admission | - | 1.31±0.75 |
|  |  |  |  | 51 | - | At ICU discharge | - | 0.74±0.54 |
|  |  |  |  | 51 | - | 1 month after ICU discharge | - | 0.5±0.38 |
| Young, 2005 [10] | HADS | 0-21 | ≥ 8 | 20 | - | 3 months after ICU discharge | 50% | - |
|  |  |  | ≥ 11 |  |  |  | 35% | - |
| HADS: Hospital Anxiety and Depression Scale  ICU: Intensive care unit  BSI: Brief Symptom Inventory | | |  |  |  |  |  |  |

1. Anderson WG, Arnold RM, Angus DC, Bryce CL. Posttraumatic stress and complicated grief in family members of patients in the intensive care unit. Journal of general internal medicine. 2008;23(11):1871-6. doi:10.1007/s11606-008-0770-2.

2. Azoulay E, Pochard F, Kentish-Barnes N, Chevret S, Aboab J, Adrie C et al. Risk of post-traumatic stress symptoms in family members of intensive care unit patients. American journal of respiratory and critical care medicine. 2005;171(9):987-94. doi:10.1164/rccm.200409-1295OC.

3. de Miranda S, Pochard F, Chaize M, Megarbane B, Cuvelier A, Bele N et al. Postintensive care unit psychological burden in patients with chronic obstructive pulmonary disease and informal caregivers: A multicenter study. Critical care medicine. 2011;39(1):112-8. doi:10.1097/CCM.0b013e3181feb824.

4. Garrouste-Orgeas M, Coquet I, Perier A, Timsit JF, Pochard F, Lancrin F et al. Impact of an intensive care unit diary on psychological distress in patients and relatives*. Critical care medicine. 2012;40(7):2033-40. doi:10.1097/CCM.0b013e31824e1b43.

5. Jones C, Skirrow P, Griffiths RD, Humphris G, Ingleby S, Eddleston J et al. Post-traumatic stress disorder-related symptoms in relatives of patients following intensive care. Intensive care medicine. 2004;30(3):456-60. doi:10.1007/s00134-003-2149-5.

6. Lemiale V, Kentish-Barnes N, Chaize M, Aboab J, Adrie C, Annane D et al. Health-related quality of life in family members of intensive care unit patients. Journal of palliative medicine. 2010;13(9):1131-7. doi:10.1089/jpm.2010.0109.

7. McAdam JL, Fontaine DK, White DB, Dracup KA, Puntillo KA. Psychological symptoms of family members of high-risk intensive care unit patients. American journal of critical care : an official publication, American Association of Critical-Care Nurses. 2012;21(6):386-93; quiz 94. doi:10.4037/ajcc2012582.

8. Myhren H, Ekeberg O, Langen I, Stokland O. Emotional strain, communication, and satisfaction of family members in the intensive care unit compared with expectations of the medical staff: experiences from a Norwegian University Hospital. Intensive care medicine. 2004;30(9):1791-8. doi:10.1007/s00134-004-2375-5.

9. Wartella JE, Auerbach SM, Ward KR. Emotional distress, coping and adjustment in family members of neuroscience intensive care unit patients. Journal of psychosomatic research. 2009;66(6):503-9. doi:10.1016/j.jpsychores.2008.12.005.

10. Young E, Eddleston J, Ingleby S, Streets J, McJanet L, Wang M et al. Returning home after intensive care: a comparison of symptoms of anxiety and depression in ICU and elective cardiac surgery patients and their relatives. Intensive care medicine. 2005;31(1):86-91. doi:10.1007/s00134-004-2495-y.
