# Supplementary material for: Reported burden on informal caregivers of ICU survivors: a literature review
Source: Crit Care. 2016 Jan 21;20:16. doi: 10.1186/s13054-016-1185-9 (PMC4721206; doi:10.1186/s13054-016-1185-9)
Supplement: Supplementary file 5 — Post-traumatic stress: assessment tools, time points and outcomes measures for caregivers for quantitative studies. (DOC 77 kb) [file 13054_2016_1185_MOESM5_ESM.doc]

| **Additional table 5.** Post-traumatic stress: Assessment tools, time points and outcomes measures for caregivers for quantitative studies | | | | | | | | |
| --- | --- | --- | --- | --- | --- | --- | --- | --- |
| Author, year | Assessment tool | Score range | Cut-off score | n | Subgroup | Time of measurement | Prevalence % | Mean±SD |
| Anderson, 2008 [1] | IES | 0-75 | >30 | 34 | - | 6 months after enrolment | 35% | 25±19 |
| Azoulay, 2005 [2] | IES | 0-75 | >30 | 284 | - | 3 months after ICU discharge or death | 33.1% | 22 (11-34)*^a^* |
| De Miranda, 2011 [3] | IES-R | 0-88 | ≥22 | 47 | - | 3 months after ICU discharge | 29.8% | - |
| Dithole, 2013 [4] | PCL-S | 17-85 | >44 | 28 | - | 6 months after ICU discharge | 57.1% | - |
| Garrouste-Orgeas, 2012 [5] | IES-R | 0-88 | ≥22 | 40 | Pre-diary | 12 months after ICU discharge | 80% | 32.7±12.9 |
|  |  |  |  | 41 | Diary |  | 31.7% | 21.6±10.7 |
|  |  |  |  | 34 | Post-diary |  | 67.6% | 29.8±14.5 |
| Jones, 2004 [6] | IES | 0-75 | >19 | 50 | Rehabilitation | 2 months after ICU discharge | 53% | 23.6±19 |
|  |  |  |  | 40 | Control |  | 63% | 25±18.2 |
|  |  |  |  | 47 | Rehabilitation | 6 months after ICU discharge | 38% | 21.8±18.5 |
|  |  |  |  | 37 | Control |  | 55% | 27±20.86 |
| Jones, 2012 [7] | PTSS-14 | 14-98 | - | 15 | Intervention | 1 month after ICU discharge | - | 31.2±15.3 |
|  |  |  |  | 15 | Control |  | - | 32.1±15.9 |
|  |  |  |  | 15 | Intervention | 3 months after ICU discharge | - | 21.7±8.3 |
|  |  |  |  | 15 | Control |  | - | 30.9±15.2 |
| McAdam, 2012 [8] | IES-R | 0-88 | Mean score ≥ 1.5 | 74 | - | During ICU stay | 56.8% | 1.74±0.88 |
|  |  |  |  | 41 | - | 3 months after ICU discharge or death | 42% | 1.27±0.86 |
| ICU: Intensive care unit  IES: Impact of Event Scale  IES-R: Impact of Event Scale-Revised  PCL-S: PTSD Checklist-Specific scale  PTSS-14: Post-Traumatic Stress Syndrome-14 scale | | | *^a^* Median (IQR) | |  |  |  |  |
|  |  |  |  |  |  |  |  |  |
|  |  |  |  |  |  |  |  |  |
|  |  |  |  |  |  |  |  |  |

1. Anderson WG, Arnold RM, Angus DC, Bryce CL. Posttraumatic stress and complicated grief in family members of patients in the intensive care unit. Journal of general internal medicine. 2008;23(11):1871-6. doi:10.1007/s11606-008-0770-2.

2. Azoulay E, Pochard F, Kentish-Barnes N, Chevret S, Aboab J, Adrie C et al. Risk of post-traumatic stress symptoms in family members of intensive care unit patients. American journal of respiratory and critical care medicine. 2005;171(9):987-94. doi:10.1164/rccm.200409-1295OC.

3. de Miranda S, Pochard F, Chaize M, Megarbane B, Cuvelier A, Bele N et al. Postintensive care unit psychological burden in patients with chronic obstructive pulmonary disease and informal caregivers: A multicenter study. Critical care medicine. 2011;39(1):112-8. doi:10.1097/CCM.0b013e3181feb824.

4. Dithole K, Thupayagale-Tshweneagae G, Mgutshini T. Posttraumatic stress disorder among spouses of patients discharged from the intensive care unit after six months. Issues in mental health nursing. 2013;34(1):30-5. doi:10.3109/01612840.2012.715235.

5. Garrouste-Orgeas M, Coquet I, Perier A, Timsit JF, Pochard F, Lancrin F et al. Impact of an intensive care unit diary on psychological distress in patients and relatives*. Critical care medicine. 2012;40(7):2033-40. doi:10.1097/CCM.0b013e31824e1b43.

6. Jones C, Skirrow P, Griffiths RD, Humphris G, Ingleby S, Eddleston J et al. Post-traumatic stress disorder-related symptoms in relatives of patients following intensive care. Intensive care medicine. 2004;30(3):456-60. doi:10.1007/s00134-003-2149-5.

7. Jones C, Backman C, Griffiths RD. Intensive care diaries and relatives' symptoms of posttraumatic stress disorder after critical illness: a pilot study. American journal of critical care : an official publication, American Association of Critical-Care Nurses. 2012;21(3):172-6. doi:10.4037/ajcc2012569.

8. McAdam JL, Fontaine DK, White DB, Dracup KA, Puntillo KA. Psychological symptoms of family members of high-risk intensive care unit patients. American journal of critical care : an official publication, American Association of Critical-Care Nurses. 2012;21(6):386-93; quiz 94. doi:10.4037/ajcc2012582.
