# Supplementary material for: Reported burden on informal caregivers of ICU survivors: a literature review
Source: Crit Care. 2016 Jan 21;20:16. doi: 10.1186/s13054-016-1185-9 (PMC4721206; doi:10.1186/s13054-016-1185-9)
Supplement: Supplementary file 6 — Health-related quality of life: assessment tools, time points and outcomes measures for caregivers for quantitative studies. (DOC 80 kb) [file 13054_2016_1185_MOESM6_ESM.doc]

| **Additional table 6.** Health related quality of life: Assessment tools, time points and outcomes measures for caregivers for quantitative studies | | | | | | | | |
| --- | --- | --- | --- | --- | --- | --- | --- | --- |
| Author, year | Assessment tool | Score range | Cut-off score | n | Subgroup | Time of measurement | Prevalence % | Mean±SD |
| Azoulay, 2005 [1] | SF-36 | 0-100 | - | 284 | - | 3 months after ICU discharge or death | - | - |
| Bayen, 2013 [2] | SF-36 - PCS  SF-36 - MCS | 0-100 | - | 66 | - | 1 year after injury | - | 48.3±8.9 |
|  |  | 0-100 | - | 66 | - |  | - | 36.7±11.7 |
| Cameron, 2006 [3] | SF-36 - Physical functioning  SF-36 - Role physical  SF-36 - Bodily pain  SF-36 - General health  SF-36 - Vitality  SF-36 - Social functioning  SF-36 - Role emotional  SF-36 - Mental health | 0-100  0-100  0-100  0-100  0-100  0-100  0-100  0-100 | -  -  -  -  -  -  -  - | 47 | - | On average 23 months after hospital discharge |  | 55 (28-80)*^a^*  67 (0-100)*^a^*  62 (32-100)*^a^* |
|  |  |  |  |  |  |  |  |  |
|  |  |  |  |  |  |  |  | 52 (35-72)*^a^* |
|  |  |  |  |  |  |  |  | 50 (30-70)*^a^* |
|  |  |  |  |  |  |  |  | 75 (38-100)*^a^* |
|  |  |  |  |  |  |  |  | 67 (0-100)*^a^* |
|  |  |  |  |  |  |  |  | 76 (47-88)*^a^* |
| Douglas, 2003 [4] | 5-point Likert scale | 1-5 | ≤ 2 | 135 | - | At hospital discharge | 19.0% | 3.74±1.0 |
|  |  |  |  | 77 | - | 6 months after hospital discharge | 26.8% | 3.29±1.1 |
| Douglas, 2005 [5] | SF-8 - PCS | 0-100 | - | 211 | Experimental | At hospital discharge | - | 52.9±7.7 |
|  |  |  |  | 79 | Control |  | - | 51.9±8.2 |
|  |  |  |  | 163 | Experimental | 2 months after hospital discharge | - | 51.3±9.4 |
|  |  |  |  | 48 | Control |  | - | 51.5 ±10.2 |
|  | SF-8 - MCS | 0-100 | - | 163 | Experimental | At hospital discharge | - | 45.2±12.0 |
|  |  |  |  | 48 | Control |  | - | 45.9±11.8 |
|  |  |  |  | 163 | Experimental | 2 months after hospital discharge | - | 44.4±12.7 |
|  |  |  |  | 48 | Control |  | - | 46.3 ±11.2 |
| Douglas, 2010 [6] | 5-point Likert scale | 1-5 | ≤ 2 | 252 | White | ICU admission | 47.6% | 3.8±1.4 |
|  |  |  |  | 118 | Nonwhite |  | 59.5% | 3.4±1.5 |
|  |  |  |  | 193 | White | 2 months after hospital discharge | 66.1%  86.0% | 3.3±1.4 |
|  |  |  |  | 84 | Nonwhite |  |  | 2.9±1.4 |
| Lemiale, 2010 [7] | SF-36 - PCS  SF-36 - MCS   - role emotional - social functioning - vitality - mental health | 0-100  0-100 | - | 284 | - | 3 months after ICU discharge or death | - | 89 (66-94)*^a^*  -  66 (50-80)*^a^*  70 (60-90)*^a^*  60 (45-70)*^a^*  64.6 (53.2-77.7)*^a^* |
| ICU: Intensive care unit  MCS: Mental component summary  PCS: Physical component summary  SF-36: Short Form-36  SF-8: Short Form-8 | | | *^a^* Median (IQR) | |  |  |  |  |

1. Azoulay E, Pochard F, Kentish-Barnes N, Chevret S, Aboab J, Adrie C et al. Risk of post-traumatic stress symptoms in family members of intensive care unit patients. American journal of respiratory and critical care medicine. 2005;171(9):987-94. doi:10.1164/rccm.200409-1295OC.

2. Bayen E, Pradat-Diehl P, Jourdan C, Ghout I, Bosserelle V, Azerad S et al. Predictors of informal care burden 1 year after a severe traumatic brain injury: results from the PariS-TBI study. The Journal of head trauma rehabilitation. 2013;28(6):408-18. doi:10.1097/HTR.0b013e31825413cf.

3. Cameron JI, Herridge MS, Tansey CM, McAndrews MP, Cheung AM. Well-being in informal caregivers of survivors of acute respiratory distress syndrome. Critical care medicine. 2006;34(1):81-6.

4. Douglas SL, Daly BJ. Caregivers of long-term ventilator patients: physical and psychological outcomes. Chest. 2003;123(4):1073-81.

5. Douglas SL, Daly BJ, Kelley CG, O'Toole E, Montenegro H. Impact of a disease management program upon caregivers of chronically critically ill patients. Chest. 2005;128(6):3925-36. doi:10.1378/chest.128.6.3925.

6. Douglas SL, Daly BJ, O'Toole E, Hickman RL, Jr. Depression among white and nonwhite caregivers of the chronically critically ill. Journal of critical care. 2010;25(2):364 e11-9. doi:10.1016/j.jcrc.2009.09.004.

7. Lemiale V, Kentish-Barnes N, Chaize M, Aboab J, Adrie C, Annane D et al. Health-related quality of life in family members of intensive care unit patients. Journal of palliative medicine. 2010;13(9):1131-7. doi:10.1089/jpm.2010.0109.
