# Supplementary material for: Reported burden on informal caregivers of ICU survivors: a literature review
Source: Crit Care. 2016 Jan 21;20:16. doi: 10.1186/s13054-016-1185-9 (PMC4721206; doi:10.1186/s13054-016-1185-9)
Supplement: Supplementary file 7 — Lifestyle interference: assessment tools, time points and outcomes measures for caregivers for quantitative studies. (DOC 65 kb) [file 13054_2016_1185_MOESM7_ESM.doc]

| **Additional table 7.** Lifestyle interference: Assessment tools, time points and outcomes measures for caregivers for quantitative studies | | | | | | | | |
| --- | --- | --- | --- | --- | --- | --- | --- | --- |
| Author, year | Assessment tool | Score range | Cut-off score | n | Subgroup | Follow-up | Prevalence % | Mean±SD |
| Cameron, 2006 [1] | CIS | 0-84 | - | 47 | - | On average 23 months after hospital discharge | - | 20.3±24.0 |
| Choi, 2011 [2] | CRF | 11-44 | - | 69 | - | 1 month after ICU discharge | - | 23±8.3 |
|  |  |  |  | 69 | - | 6 months after ICU discharge | - | 19.4±8.6 |
| Douglas, 2003 [3] | “objective indicator” portion of the “objective and subjective burden” tool | 9-45 | - | 135 | - | At hospital discharge | - | 32.97±4.98 |
|  |  |  |  | 77 | - | 6 months after hospital discharge | - | 31.1±6.5 |
| Im, 2004 [4] | CRF | 11-44 | - | 115 | - | 2 months following the onset of prolonged MV | - | 22.1±8.5 |
| Swoboda, 2002 [5] | FIS - Quit other activities to care for family | Yes/no | Yes | 95 | - | Situation 2 weeks before ICU admission | 44.2% | - |
|  |  |  |  | 69 | - | 1 month after ICU admission | 84.5% | - |
|  |  |  |  | 61 | - | 3 months after ICU admission | 63.9% | - |
|  |  |  |  | 53 | - | 6 months after ICU admission | 50.9% | - |
|  |  |  |  | 52 | - | 12 months after ICU admission | 45.8% | - |
| Van Pelt, 2007 [6] | ARS | 11-44 | - | 115 | - | 2 months after MV initiation | - | 22.1±8.5 |
|  |  |  |  | 107 | - | 6 months after MV initiation | - | 20.5±8.4 |
|  |  |  |  | 92 | - | 12 months after MV initiation | - | 20.0±8.4 |
| Van Pelt, 2010 [7] | ARS | 11-44 | - | 48 | - | 2 months after MV initiation | - | 22.3±8.7 |
|  |  |  |  | 48 |  | 6 months after MV initiation | - | 19.7±8.1 |
|  |  |  |  | 48 |  | 12 months after MV initiation | - | 19.4±8.0 |

| ARS: Activity Restriction Scale  CIS: Caregiving Impact Scale  CRF: Changes in Role Function scale  FIS: Family Impact Survey  ICU: Intesive care unit  MV: Mechanical ventilation |
| --- |

1. Cameron JI, Herridge MS, Tansey CM, McAndrews MP, Cheung AM. Well-being in informal caregivers of survivors of acute respiratory distress syndrome. Critical care medicine. 2006;34(1):81-6.

2. Choi J, Donahoe MP, Zullo TG, Hoffman LA. Caregivers of the chronically critically ill after discharge from the intensive care unit: six months' experience. American journal of critical care : an official publication, American Association of Critical-Care Nurses. 2011;20(1):12-22; quiz 3. doi:10.4037/ajcc2011243.

3. Douglas SL, Daly BJ. Caregivers of long-term ventilator patients: physical and psychological outcomes. Chest. 2003;123(4):1073-81.

4. Im K, Belle SH, Schulz R, Mendelsohn AB, Chelluri L, Investigators Q-M. Prevalence and outcomes of caregiving after prolonged (> or =48 hours) mechanical ventilation in the ICU. Chest. 2004;125(2):597-606.

5. Swoboda SM, Lipsett PA. Impact of a prolonged surgical critical illness on patients' families. American journal of critical care : an official publication, American Association of Critical-Care Nurses. 2002;11(5):459-66.

6. Van Pelt DC, Milbrandt EB, Qin L, Weissfeld LA, Rotondi AJ, Schulz R et al. Informal caregiver burden among survivors of prolonged mechanical ventilation. American journal of respiratory and critical care medicine. 2007;175(2):167-73. doi:10.1164/rccm.200604-493OC.

7. Van Pelt DC, Schulz R, Chelluri L, Pinsky MR. Patient-specific, time-varying predictors of post-ICU informal caregiver burden: the caregiver outcomes after ICU discharge project. Chest. 2010;137(1):88-94. doi:10.1378/chest.09-0795.
